# Chondrosarcoma evaluation using hematein-based x-ray staining and high-resolution 3D micro-CT: a feasibility study

**DOI:** 10.1186/s41747-024-00454-0

**Published:** 2024-05-13

**Authors:** Alexandra S. Gersing, Melanie A. Kimm, Christine Bollwein, Patrick Ilg, Carolin Mogler, Felix G. Gassert, Georg C. Feuerriegel, Carolin Knebel, Klaus Woertler, Daniela Pfeiffer, Madleen Busse, Franz Pfeiffer

**Affiliations:** 1 Department of Diagnostic and Interventional Radiology, School of Medicine, Klinikum rechts der Isar, Technical University of Munich, Ismaningerstr. 22, Munich, 81675 Germany; 2 Department of Neuroradiology, LMU University Hospital, LMU Munich, Munich, Germany; 3 Department of Radiology, LMU University Hospital, LMU Munich, Marchioninistr. 15, Munich, 81377 Germany; 4 Institute of Pathology, School of Medicine, Technical University of Munich, Trogerstrasse 18, Munich, 81675 Germany; 5 Munich Institute of Biomedical Engineering, Technical University of Munich, Garching, 85748 Germany; 6 Chair of Biomedical Physics, Department of Physics, School of Natural Sciences, Technical University of Munich, Garching, 85748 Germany; 7 Department of Orthopedics and Sports Orthopedics, Technical University of Munich, Ismaninger Str. 22, Munich, 81675 Germany; 8 Musculoskeletal Radiology Section, Klinikum rechts der Isar, School of Medicine, Technical University of Munich, Munich, 81675 Germany; 9 Munich Institute for Advanced Study, Technical University of Munich, Garching, 85748 Germany

**Keywords:** Bone neoplasms, Chondrosarcoma, Hematein, Staining and labeling, X-ray microtomography

## Abstract

**Background:**

Chondrosarcomas are rare malignant bone tumors diagnosed by analyzing radiological images and histology of tissue biopsies and evaluating features such as matrix calcification, cortical destruction, trabecular penetration, and tumor cell entrapment.

**Methods:**

We retrospectively analyzed 16 cartilaginous tumor tissue samples from three patients (51-, 54-, and 70-year-old) diagnosed with a dedifferentiated chondrosarcoma at the femur, a moderately differentiated chondrosarcoma in the pelvis, and a predominantly moderately differentiated chondrosarcoma at the scapula, respectively. We combined a hematein-based x-ray staining with high-resolution three-dimensional (3D) microscopic x-ray computed tomography (micro-CT) for nondestructive 3D tumor assessment and tumor margin evaluation.

**Results:**

We detected trabecular entrapment on 3D micro-CT images and followed bone destruction throughout the volume. In addition to staining cell nuclei, hematein-based staining also improved the visualization of the tumor matrix, allowing for the distinction between the tumor and the bone marrow cavity. The hematein-based staining did not interfere with further conventional histology. There was a 5.97 ± 7.17% difference between the relative tumor area measured using micro-CT and histopathology (*p* = 0.806) (Pearson correlation coefficient *r* = 0.92, *p* = 0.009). Signal intensity in the tumor matrix (4.85 ± 2.94) was significantly higher in the stained samples compared to the unstained counterparts (1.92 ± 0.11, *p* = 0.002).

**Conclusions:**

Using nondestructive 3D micro-CT, the simultaneous visualization of radiological and histopathological features is feasible.

**Relevance statement:**

3D micro-CT data supports modern radiological and histopathological investigations of human bone tumor specimens. It has the potential for being an integrative part of clinical preoperative diagnostics.

**Key points:**

• Matrix calcifications are a relevant diagnostic feature of bone tumors.

• Micro-CT detects all clinically diagnostic relevant features of x-ray-stained chondrosarcoma.

• Micro-CT has the potential to be an integrative part of clinical diagnostics.

**Graphical Abstract:**

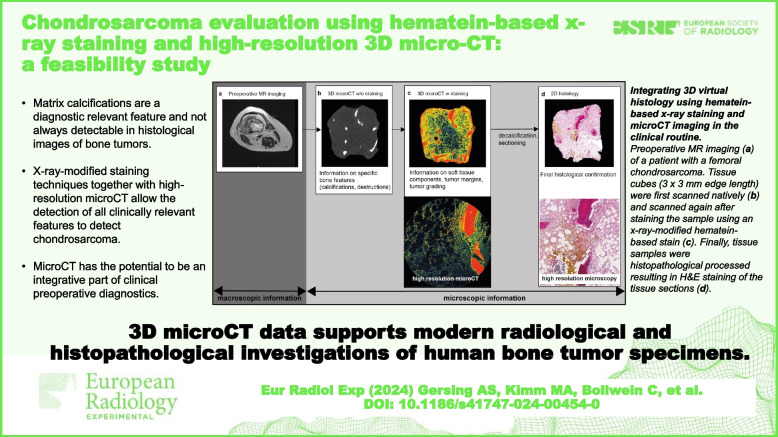

**Supplementary Information:**

The online version contains supplementary material available at 10.1186/s41747-024-00454-0.

## Background

Central cartilage tumors are malignant neoplasms with chondrosarcomas representing the most common subtype [1–4], characterized by abundant cartilaginous matrix with atypical chondrocytes. The World Health Organization Classification of Tumors of Soft Tissue and Bone classifies chondrogenic bone tumors into benign (*e.g.*, enchondromas), intermediate (*e.g.*, atypical cartilaginous tumors), and malignant (grade 1, 2, or 3, periosteal, clear cell, mesenchymal, and dedifferentiated chondrosarcoma) [5]. The grade classification of chondrosarcomas is based on chondrocyte cellularity and nuclear pleomorphism as well as presence or absence of mitoses or necrosis [6]. In grade 3 chondrosarcomas, the cells at the periphery of the tumor lobules become less differentiated and display a spindle cell morphology. The term atypical cartilaginous tumor is used for well-differentiated aggressive cartilaginous tumors located in the appendicular skeleton (long and short tubular bones). In contrast, well-differentiated cartilaginous tumors in the axial skeleton (flat bones, including the pelvis, scapula, and skull base) are called grade 1 chondrosarcomas [7].

To distinguish the different types of chondrogenic bone tumors is challenging, and therapeutic decisions depend on both histopathological and radiological features [2]. Preexisting bony trabecula may be completely surrounded by cartilaginous tumor tissue, a crucial diagnostic feature in order to differentiate chondrosarcomas and atypical cartilaginous tumors from benign cartilaginous tumors [8–10]. For exact diagnosis, histopathology of selected regions of the tumor and adjacent tissue need to be analyzed. To that aim, samples first need to be decalcified which removes minerals from the bone and existing tumoral calcifications often disappear after this step. This is a disadvantage as matrix calcifications are an important criterion in the differential diagnosis of bone tumors.

In this regard, an important benefit of x-ray microscopic computed tomography (micro-CT) imaging is that for micro-CT imaging bone tumor tissue must not undergo decalcification prior to imaging. Another point is the conversion of 3D tissue samples into two-dimensional slices during the standard histopathological workflow that irrevocably entails loss of 3D information. For the characterization of chondrosarcomas, information on 3D tumor tissue architecture and cellular density would be very useful, especially for the identification of trabecular entrapment by tumor tissue. Furthermore, the visualization of the tumor margins after tumor excision is important information used to confirm the complete resection of the tumor.

X-ray micro-CT is a nondestructive 3D technology with an examination volume ranging from cubic millimeters up to a few cubic centimeters. This range is limited on both ends by the desired resolution and the technological setup, *e.g.*, source-spot size and optical elements [11]. Further sample size limitations arise due to the high attenuation of hard tissue. Overall, the maximum specimen size determines the resolution, meaning that the highest spatial resolution can be achieved in specimens that are smaller in size. To overcome low attenuation of soft tissue which hampered the use of micro-CT imaging in the field of 3D virtual histology, contrast agent-based x-ray methods were developed [12–15]. The current micro-CT submicron resolution is comparable to conventional histology [16] and authors have shown for bone specimens that the microstructure of trabecular bone can be displayed and evaluated using 3D micro-CT [17, 18]. However, information about bone tumor extension and trabecular entrapment can only be collected by staining the specimen. An x-ray tailored hematein-based stain was previously developed in which high-resolution 3D soft tissue visualization has been obtained using micro-CT [12]. Since this stain specifically visualizes cell nuclei and, to a lower grade, cytoplasmic proteins, it can be used to visualize trabecular destruction in addition to tumor margin evaluation by micro-CT. This would represent a significant improvement over unstained samples in terms of micro-CT data and thus diagnostics of bone tumor specimens in addition to histopathological examinations.

To this aim, we have established a workflow that could be incorporated into everyday clinical practice in the future with micro-CT imaging being performed upstream of histology and which does not impede with histopathological procedures. The initial assessment of the micro-CT data could take place in parallel to the histological processing of the sample leading to the final diagnosis. This study aimed to evaluate the feasibility of the hematein-based x-ray staining method in combination with high-resolution micro-CT for nondestructive 3D visualization of the diagnostically relevant features of the tumor architecture and tumor margins of chondrosarcomas and its potential as future clinical diagnostic tool.

## Methods

### Study design

The study was performed at the University hospital of the Technical University of Munich, Germany, in a certified orthopedic tumor center very experienced with diagnostics and treatment of sarcomas. All samples were resection specimens obtained from the Department of Pathology, Technical University of Munich, between July 2020 and June 2022. Ethical approval was obtained for this retrospective study (ethics committee of the Technical University of Munich, 392/20 S-KH).

Inclusion criteria were as follows: (1) complete preoperative imaging performed; (2) surgical resection and histopathological analysis performed at the orthopedic tumor center at which this study was performed; and (3) interdisciplinary diagnosis performed by the tumor board, consisting of specialized radiologists, pathologists, and orthopedic surgeons. The diagnosis needed to be completed by the time the micro-CT analysis started.

In total, 28 cartilaginous tumors were evaluated for inclusion; 13 were diagnosed as enchondroma and 8 as atypical cartilaginous tumor. Seven cartilaginous tumors received the diagnosis of chondrosarcoma (grade 2 or 3 or dedifferentiated chondrosarcoma) and were therefore eligible for study inclusion. Of them, recurrent chondrosarcomas (*n* = 2) were excluded due to tissue damage after therapy. Moreover, the access to preoperative imaging datasets for the patient selection was not possible if preoperative imaging had been performed in a different institution (*n* = 1), if imaging datasets were incomplete (*n* = 1), or if the samples were damaged and therefore a matching was not feasible (*n* = 1), and specimens were excluded from the study. Therefore, specimen samples of only 3 patients with a chondrosarcoma were included.

Patient A (70 years old) was diagnosed with a dedifferentiated chondrosarcoma (and grade 2 chondrosarcoma components) located at the right femur, patient B (51 years old) was diagnosed with a moderately differentiated chondrosarcoma (grade 2) located in the pelvis, and patient C (54 years old) was diagnosed with a predominantly moderately differentiated chondrosarcoma (grade 3) located at the right scapula. None of the patients had metastases at the time of diagnosis.

### Workflow

**Fig. 1:**
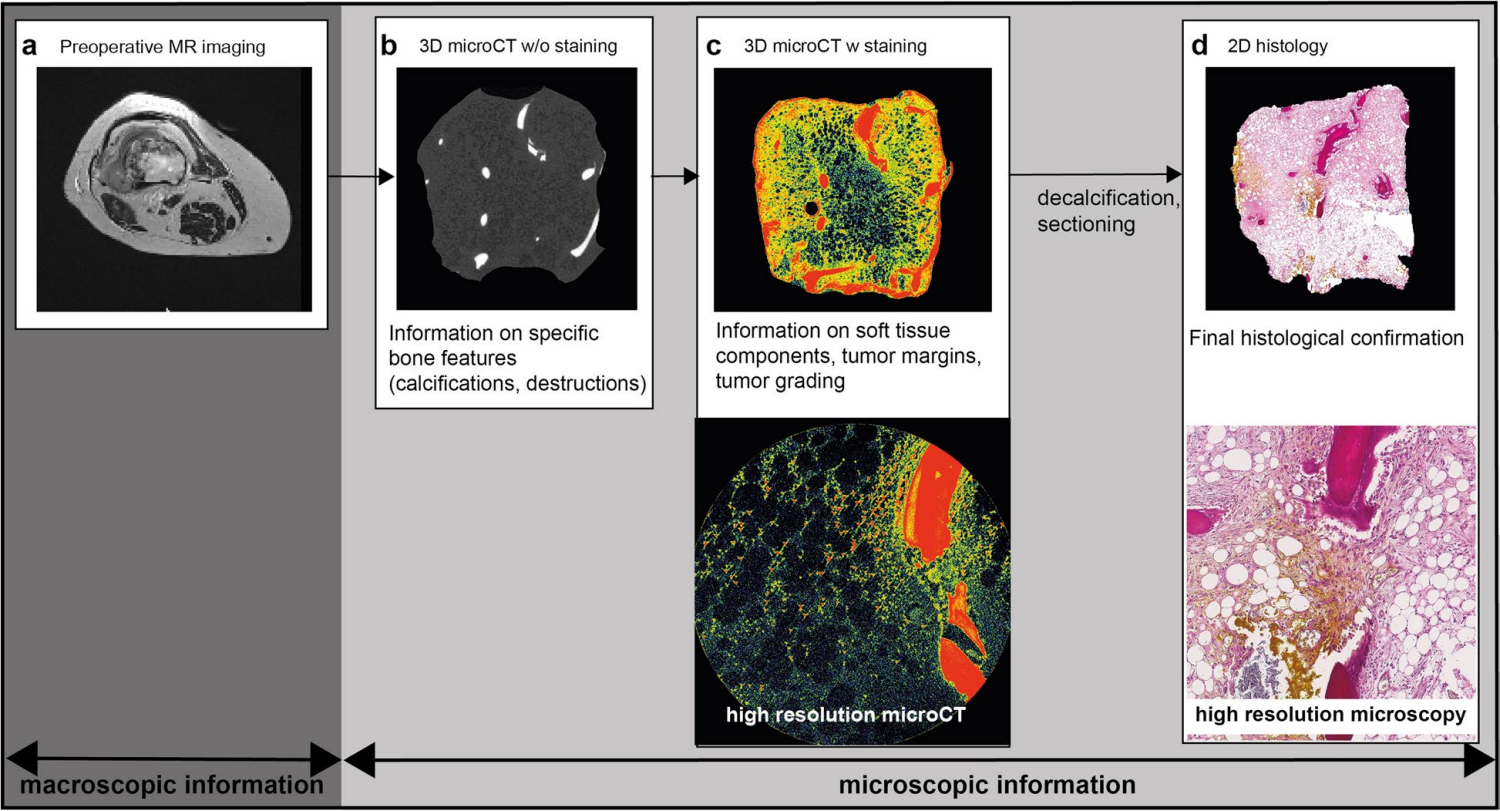
Diagnostic workflow. Representative example of a chondrosarcoma specimen undergoing three-dimensional (3D) microscopic computed tomography (micro-CT) and two-dimensional (2D) histopathological evaluation. Preoperative magnetic resonance imaging (**a**) of a patient with a femoral chondrosarcoma. Tissue cubes (3 mm × 3 mm edge length) were first scanned natively (**b**) and after staining the sample using an x-ray-modified hematein-based stain (**c**). Finally, tissue samples were histopathological processed resulting in hematoxylin and eosin staining of the tissue sections (**d**)

The established workflow was adapted to meet the clinical and pathological requirements (Fig. 1). Patients received preoperative MR imaging, and tissue biopsies were taken. The samples of each specimen were obtained from the tumor-bone transition zone in various locations as well as from the tumorous tissue and were cut into 10 mm × 10 mm × 10 mm blocks. In total, 16 samples were generated from the three patients with chondrosarcoma. Briefly, the 16 formalin-fixed (4% phosphate-buffered saline formalin solution) samples were first examined without staining using micro-CT. Afterwards, the samples were stained and reexamined using micro-CT (overview and high-resolution images) before submitting to pathology for histological analysis. Once the micro-CT workflow was established, the micro-CT scan without staining of the specimen took around 1.5 h (+ 0.5 h for transfer and preparation) and started as soon as the specimen was resected. After the first micro-CT scan, the specimen was stained for about 2 h (depending on the sample size) and rescanned using the micro-CT (again around 1.5 h). After this scan, the images were evaluated immediately. In certain unclear regions of the micro-CT images, high-resolution images were useful for the diagnosis, which took from 1.5 to 16.0 h, depending on the spatial resolution and field of view. Again, the analysis of the micro-CT was matched and analyzed immediately after the scan. Overall, the estimated timeframe for the acquisition of the micro-CT datasets ranged from 5 h to 21.5 h. The time for the qualitative evaluation of the micro-CT images varied from 0.5 to 1.5 h, depending on the complexity of the case. Volumetric analysis of the micro-CT images took a longer time.

### Hematein-based x-ray staining of tissue samples

For soft tissue visualization, a previously developed x-ray compatible tissue staining method, using a lead-hematein complex, was employed [12]. Samples were first acidified with a solution of 5% (v/v) glacial acetic acid (ThermoFisher Scientific, Waltham, MA, USA) and 95% of 4% neutral-buffered formalin (Carl Roth, Karlsruhe, Germany). Subsequently, the samples were incubated in a 0.666-M aqueous lead(II)-acetate solution (dissolved in distilled water) for 72 h on a shaking plate. After that, the samples were shortly removed to add the same volume of ripened hematein solution (0.333 M hematoxylin in ethanol) and incubated for another 72 h. The samples were taken out of the staining solution, and excess stain was removed. Following this step, the samples were washed for 24 h in 1 × DBPS (ThermoFisher Scientific, Waltham, MA, USA) and stored in a sample holder in a 70% ethanol (Carl Roth, Karlsruhe, Germany) vapor phase.

### X-ray micro-CT and image processing

For the micro-CT data, 1,601 projections were acquired over an angular range of 360° using the Xradia Versa 500 (Zeiss, Jena, Germany) at a peak voltage of 80 kV/130 kV. The × 0.39 objective and an exposure time of 1–2 s for low standard resolution and 10–20 s for high-resolution, depending on the transmission, were chosen to achieve adequate data quality of all tissue samples. Images were reconstructed with an effective voxel size of 10–20 μm (in high-resolution mode. 1.5–3.0 μm). These settings resulted in a total acquisition time of about 95 min per micro-CT scan for low resolution and about 16 h for high-resolution: about 16 h. The chosen parameters ensured optimal transmission values around 0.30 and provided a good signal-to-noise ratio with at least 5,000 counts per pixel within the field of view. The image processing of all micro-CT data sets was performed using an in-house developed Python framework. The reconstructions of the micro-CT data were performed using X-AID (Mitos GmbH, Munich, Germany). In case of strong noise, the projections were filtered with a median or gauss filter, and in case of flux instabilities, the projections were normalized to the background. All measurements were corrected for the optimal center-shift before reconstruction [14].

### Image analysis

Micro-CT stacks acquired without staining were initially aligned manually to stained micro-CT stacks, further coregistered using affine transformations and finally resampled for a voxel-wise matching. All operations were carried out using ImFusion Suite software (ImFusion GmbH, Munich, Germany). For the semiquantitative analysis, the aligned datasets were calibrated using the surrounding air (= 0) and polypropylene reaction tube (= 1) as reference materials. Three volumes of interest (VOIs) were measured per sample with each VOI having a volume of about 0.041 mm^3^. Registration and volume renderings of the micro-CT data were generated with Osirix® MD v10.0.1 software (Pixmeo SARL, Bernex, Switzerland), Horos® (version 3.3.6, www.horosproject.org), and ImFusion Suite (ImFusion GmbH, Munich,Germany). Histological images were manually matched to micro-CT images using prominent anatomical features for orientation. Color marking of tissue biopsies allowed the finding of slides along the *z*-axis.

All images were inspected visually individually and independently by a board-certified radiologist (A.S.G., 12 years of experience), an experienced radiologist (F.G.G., 5 years of experience), and a board-certified pathologist (C.B., 6 years of experience). In case of diverging evaluations (*n* = 2), a consensus reading was performed with a third board-certified musculoskeletal radiologist (K.W., 25 years of experience) and a second board-certified pathologist (17 years of experience). The main diagnostic criteria assessed for the evaluation of the micro-CT images were the following: trabecular bone morphology (presence of trabecular destruction, erosion, or encasement), presence of matrix calcifications, and tumor cellularity (high, moderate, or low). The main diagnostic criteria assessed for the evaluation of histology were the following: cellularity, chondroid matrix, myxoid matrix, and nuclear polymorphism.

For the VOI analysis for both micro-CT and histology, the relative tumor area was determined by two readers (M.A.K. and F.G.G., 20 and 5 years of experience in imaging analysis, respectively) for all samples and checked by a pathologist (C.B.). The Fiji software (version 2.4, www.fiji.sc) was used for image processing and image overlays [19].

### Histology

In order to evaluate the micro-CT images against the histological ones, the tissue samples were color-marked with tissue-marking dyes (CellPath, Newtown, Wales, UK) after micro-CT acquisition and returned to the Department of Pathology for further analysis. Prior to standard work-up, the samples were decalcified in Osteosoft® (Merck, Darmstadt, Germany). Following standard protocols, tissue samples were dehydrated, embedded into paraffin, 3-μm thick slices cut using a microtome (DM2500, Leica, Wetzlar, Germany), and mounted on Superfrost®Plus microscope slides (Carl Roth, Karlsruhe, Germany). Slides were deparaffinized and hematoxylin and eosin (H&E) stained according to the institutional standard protocol. The resulting histological images were acquired using a Leica AT2 scanner (Leica, Wetzlar, Germany) and analyzed using Aperio ImageScope software (Leica, Wetzlar, Germany).

### Reproducibility

For the VOI analysis, intra-reader and inter-reader reproducibility were analyzed by redefining the VOIs measurements of the tumor by both readers in order to calculate the relative tumor area at least four weeks later in all samples than the first reading.

### Statistics

GraphPad Prism (version 10, GraphPad Software) was used for statistical analysis. To calculate both of the intra- and inter-reader reproducibility for VOI measurements for the present study, the reproducibility error was assessed by calculating the root mean square average of the single coefficients of variation on a percentage basis, as previously reported [20]. The inter-reader intraclass correlation coefficients (ICCs) were calculated using standard guidelines [21]. ICC values below 0.50 were considered poor, values 0.50–0.75 moderate, values 0.75–0.90 good, and values above 0.90 excellent [22].

Pearson correlation coefficients analysis was calculated to determine the conformity of tumor areas in micro-CT and histological images. Normality distribution was determined by the Shapiro-Wilk-test. Data are presented as mean (± standard deviation) or median with interquartile range (IQR) according to the distribution. Unpaired data were analyzed using Mann-Whitney *U* test. A *p* value < 0.05 was considered to be significant.

## Results

### Visibility of tumor tissue

**Fig. 2:**
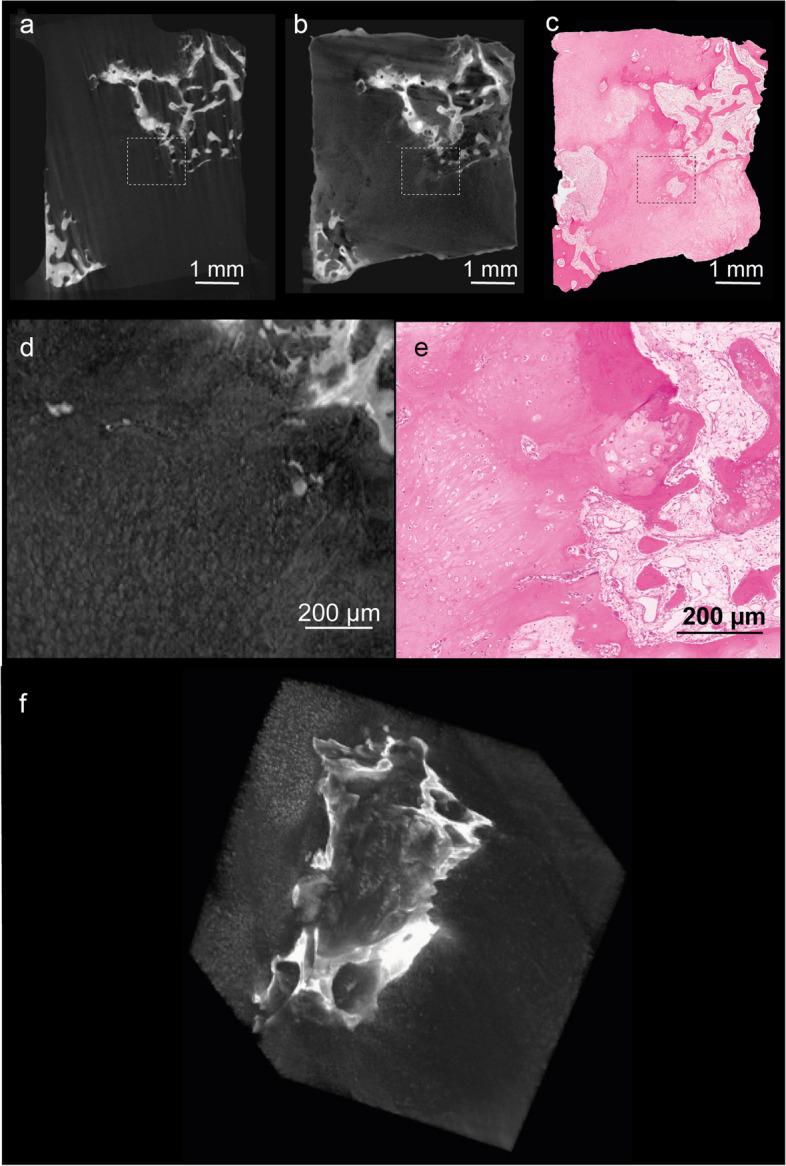
Visibility of cellular components after hematein-based x-ray staining. Representative chondrosarcoma sample showing unstained (**a**) and stained (**b**) microscopic computed tomography (micro-CT) image and corresponding histological section (**c**). High resolution micro-CT (**d**) and hematoxylin and eosin image (**e**) of the rectangle highlighted in **a**, **b**, and **c**. Cell nuclei and cytoplasm present with strong signal intensity. 3D image of the whole sample (**f**)

Tumor tissue was visible following staining and rescanning which was revised in H&E-stained sections of corresponding planes (Fig. 2a–c). Direct comparison of micro-CT data sets of unstained and stained chondrosarcoma tissue showed a noticeable difference regarding the achieved soft-tissue contrast and feature delineation as uptake of the hematein-based stain resulted in stronger signal intensity (Fig. 2a, b). In general, bone structures appear bright on micro-CT images and dark pink in histological images. The tumor component of the chondrosarcoma itself appears dark on the unstained micro-CT images (Fig. 2a), light grey in the stained micro-CT images (Fig. 2b), and light pink on the histological images (Fig. 2c), representing the cartilaginous matrix and the embedded tumor cells with varying cellular density. Contrast and feature detection was especially enhanced in the higher spatial resolution 2 × 2 × 2 μm^3^ voxel acquisitions of the chondrosarcoma samples (Fig. 2d). Notably, the 2 × 2 × 2 μm^3^ voxel data sets combined of x-ray hematein-stained samples revealed micrometer-scale intramedullary tumor tissue infiltration and allowed the distinction between cell nuclei and cytoplasm (Fig. 2d), again verified by analyzing H&E staining of the correlating layer (Fig. 2e). The 3D rendering illustrates the bone destruction and tumor tissue within the cavities simultaneously (Fig. 2f, Supplemental movie S1).

**Fig. 3:**
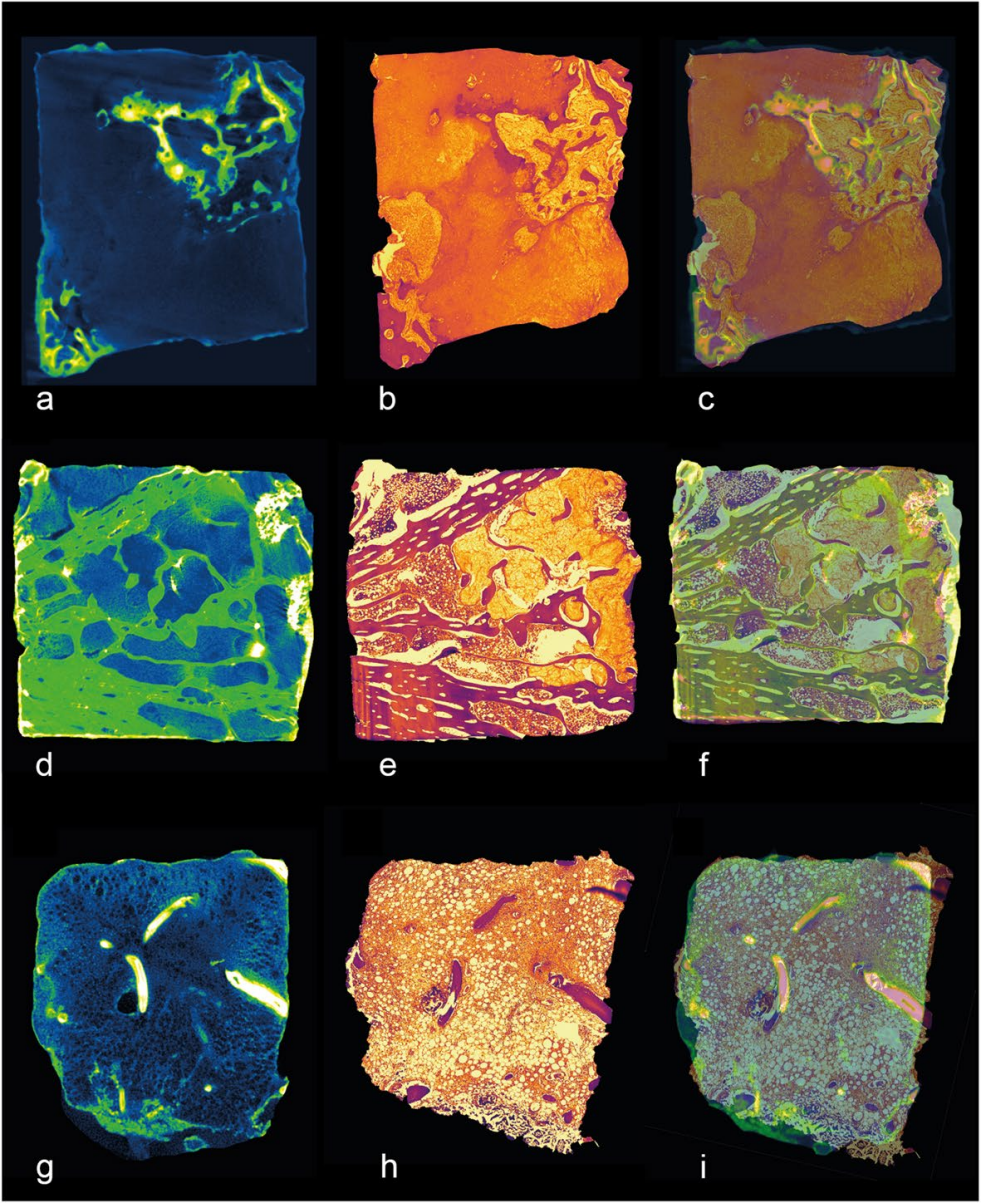
Correlation of microscopic computed tomography (micro-CT) images with histopathology. Pseudo-colored images of micro-CT stacks (**a**, **d**, **g**) and light microscopy images (**b**, **e**, **h**) were overlayed (**c**, **f**, **i**) to verify the correlative identification of specific features in both imaging modalities. We present one representative samples for each of three chondrosarcomas located: in the scapula (**a**, **b**, **c**); in the pelvis (**d**, **e**, **f**); and in the femur (**g**, **h**, **i**)

**Fig. 4:**
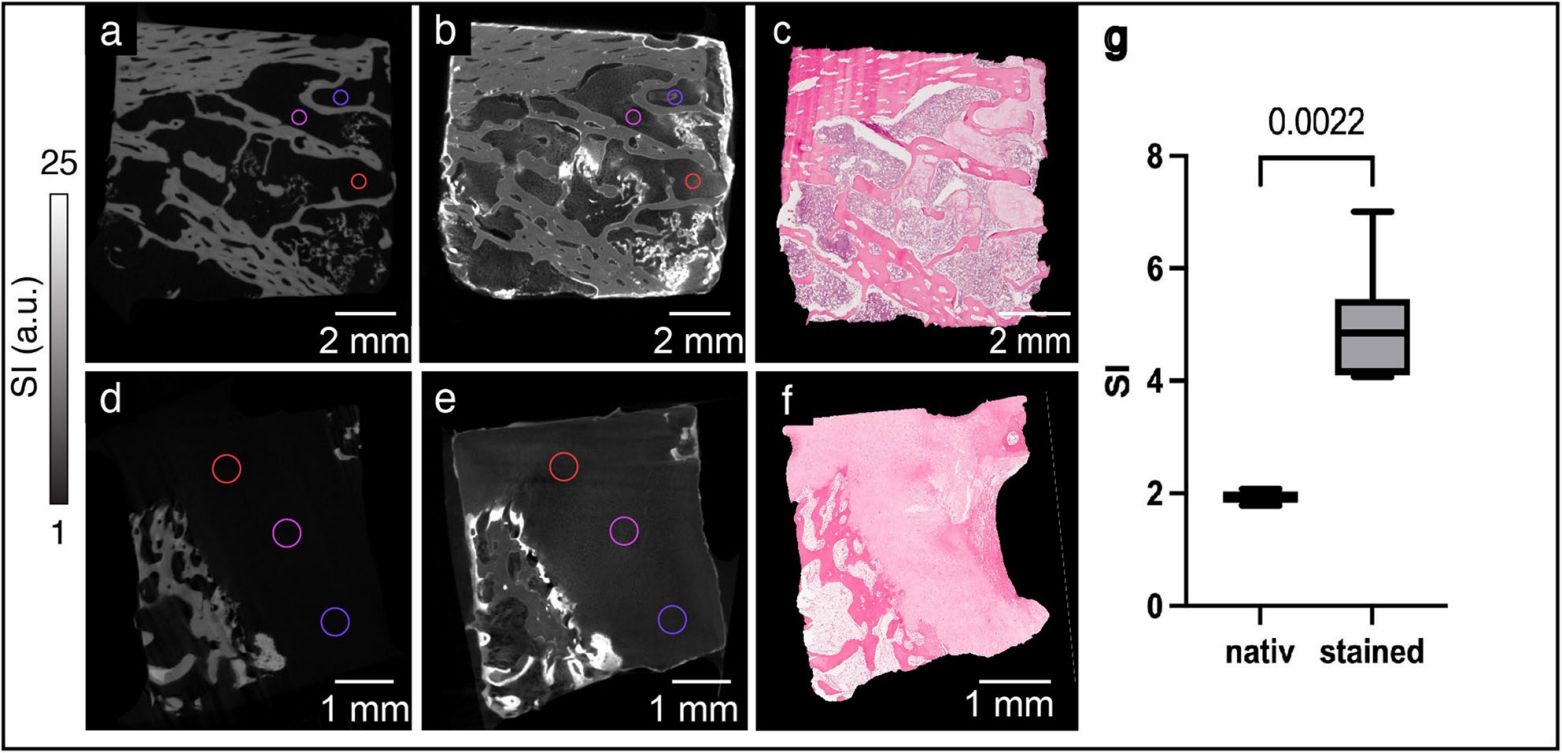
Enrichment of signal intensity in hematein-based x-ray-stained samples. Two representative chondrosarcoma samples are shown. Hematoxylin and eosin-stained sections (**c**, **f**) of the corresponding microscopic computed tomography images (**a**, **b**, **d**, **e**) to verify the positioning of the volumes of interest within unstained (**a**, **d**) and stained (**b**, **e**) tumor tissue. Signal intensities were normalized to surrounding air (= 0) and the polypropylene (= 1) of the reaction tube. Volumes of interest of the stained samples showed increased signal intensity compared to the unstained counterpart (**g**). *a.u.*, Arbitrary units; *SI*, Signal intensity

Micro-CT images of all investigated samples displayed a tissue architecture comparable to the correlating H&E-stained histological sections; thus, we used trabeculae to superimpose micro-CT and histological images (Fig. 3) and determined the relative tumor area on the images. On average, there was a 5.97 ± 7.17% difference between the relative tumor area measured using micro-CT and histopathology (*p* = 0.806), resulting in a significant Pearson correlation coefficient (*r* = 0.92, *p* = 0.009). This demonstrated the excellent agreement in the detection of tumor areas in micro-CT images and their histological counterparts. Averaged over all samples, the inter-reader reproducibility for VOI measurements between two readers was 1.93% (root mean square average of the coefficients of variation). For intra-reader reproducibility, both readers repeated the segmentations in all samples with at least 4 weeks separating the readings. The intra-reader reproducibility for overall VOI measurements of the two readers was 1.12% and 1.31%, respectively. We further analyzed the uptake of staining solution and the associated increase in signal intensity in cells of the cartilaginous tumor tissue (Fig. 4). For the semi-quantitative analysis, VOIs were placed within the tumor tissue (Fig. 4a, b, d, e) which was verified by corresponding H&E-stained sections, Fig. 4c, f). Surrounding air and the polypropylene material of the reaction tube served as reference. The signal intensity in the tumor matrix was with 4.85 (± 2.94) significantly higher in the stained samples compared to the unstained counterparts (1.92 ± 0.11, *p* = 0.002) (Fig. 4g).

### Trabecular bone morphology

**Fig. 5:**
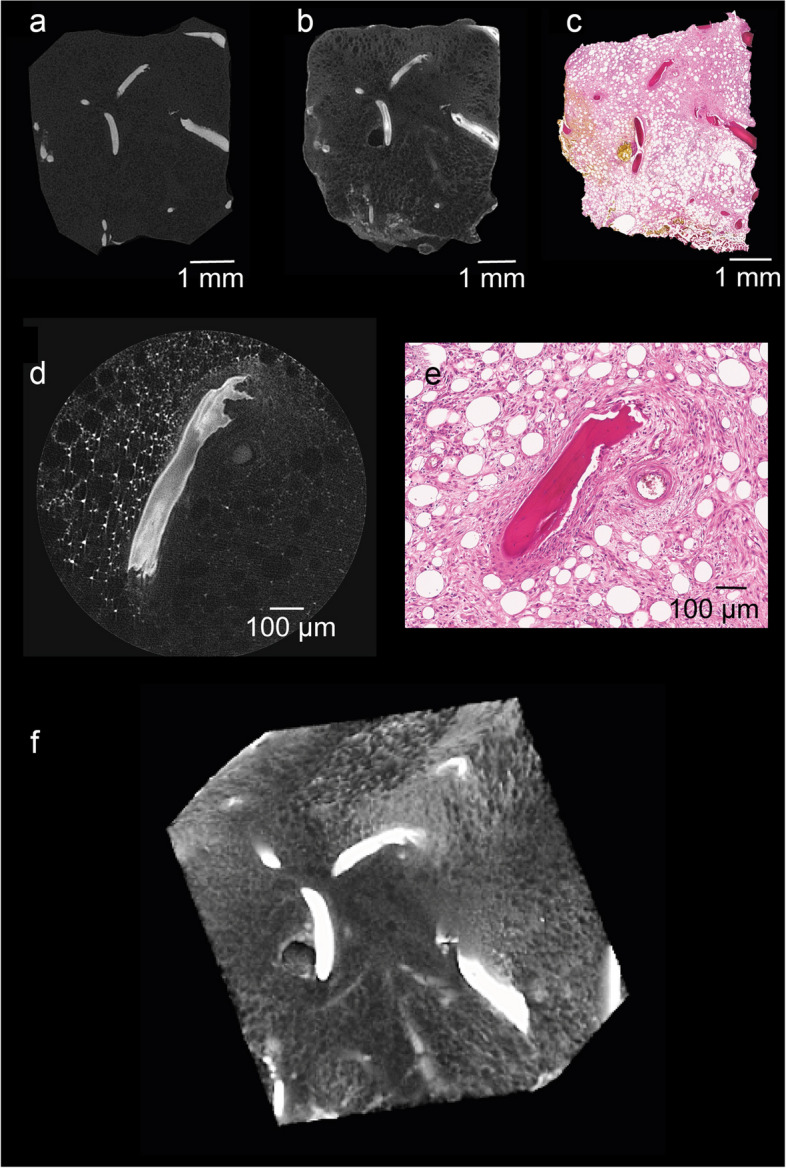
Trabecular destruction. Unstained (**a**) and hematein-based x-ray-stained overview (**b**) and high-resolution (**d**) image representing the erosion of trabecular bone. Its embedment into tumor tissue in detail in a three-dimensional reconstruction (**f**). Hematoxylin and eosin stains (**c**, **e**) of the correlating levels

In chondrosarcomas, the transition zone of bone to tumor is of particular interest due to the specific pattern of bone destruction. Therefore, we investigated the visibility of tumor tissue in hematein-based stained samples near the destructed trabeculae. The erosion and destruction of the trabeculae were already depictable in unstained samples (Fig. 5a), but the trabecular encasement through tumor tissue became only visible after staining (Fig. 5b). 3D volume rendering helped visualize the positioning of the encasement within the specimen (Fig. 5f). The high-resolution image allowed the detailed analysis of trabeculae, bone marrow fraction, and tumor tissue localization (Fig. 5d and Supplemental movie S2). H&E staining of the corresponding layers (Fig. 5c, e) confirmed features detected in micro-CT images.

**Fig. 6:**
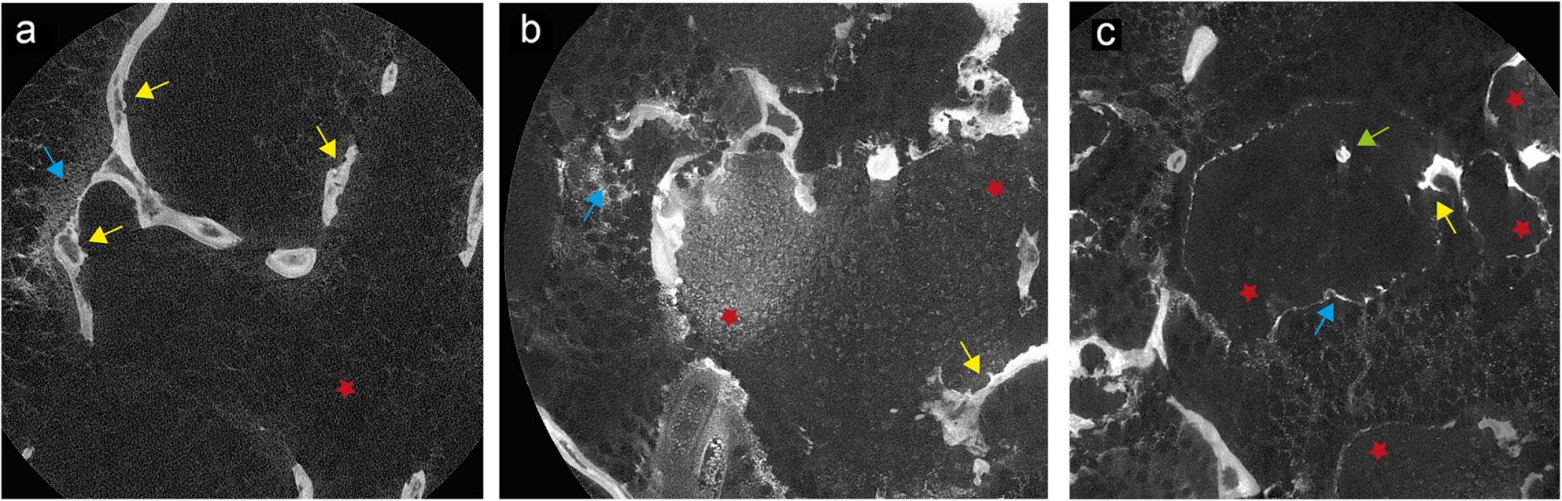
Radiological diagnostic features in stained tissue samples (**a**, **b**, **c**). Tumor cells (red stars), trabecular destruction (yellow arrows), matrix calcifications (blue arrows), and trabecular entrapment (green arrow)

Apart from the trabecular bone destruction, calcifications within the tumor matrix are important radiological features of chondrosarcomas, which were also assessed in all specimens available. Matrix calcifications were revealed as multiple cloud-like hyperdense areas (Fig. 6a–c, blue arrows) close the tumor cells. Prominent small round dots with high signal intensity represent cell nuclei, large oval dots the cytoplasm (Fig. 6a–c, red stars). The entrapment of bony trabecular by tumor tissue is a major diagnostic criteria especially for low-grade chondrosarcomas/atypical cartilaginous tumors in distinction to benign cartilaginous tumors. Here, too, we were able to detect trabecular entrapment in all stained samples (Fig. 6c, green arrow, Supplemental Fig. S1 and Supplemental movie S3). For evaluation of the micro-CT images, intra- and inter-reader intraclass correlation coefficients (ICCs) were calculated to compare each diagnostic criterion (see the “Methods” section for details). After at least 4 weeks, the image analysis was repeated once again the same way. The ICCs for intra-reader reproducibility ranged between 0.93 and 0.98 (95% confidence interval: 0.83–1.00), and the inter-reader reproducibility ranged between 0.91 and 0.96 (95% confidence interval: 0.80–1.00).

## Discussion

In this study, the hematein-based x-ray staining method in combination with high-resolution micro-CT enabled a 3D nondestructive visualization of radiological and histopathological relevant features of chondrosarcomas. Micro-CT scan with a resolution of 10 × 10 × 10 μm^3^ was performed in order to collect first information and to define VOIs of special interest, which were further studied with high-resolution (2 × 2 × 2 μm^3^). By demonstrating the clinical applicability and feasibility of nondestructive 3D micro-CT, achieved using a stain with biological relevance, we emphasize the potential of staining-based micro-CT to be integrated into clinical diagnostics.

For the assessment of the diagnostic features corresponding with histology in bone tumor diagnostics, the main prerequisites are sufficient resolution and adequate bone and tumor tissue contrast. Within the unstained sample, osseous features (bone erosion and destruction) as well as calcifications could be visualized, and the correlation of the tumor tissue volume assessed using micro-CT with the tumor tissue volume assessed using histology was highly significant.

Especially in patients with cartilaginous tumors, the final diagnosis is often made as a consensus between the pathological and radiological diagnosis. The developed protocol showed a high potential for the visualization of pathological and radiological bone tumor features. The examination of the entire resection specimen could be assessed to evaluate the resection border as well as radiological and pathological features of bone tumors and surrounding tissue. For the bone tumor tissue, the combination of hematein-based x-ray staining and micro-CT is of great interest, since this combination enabled the identification of regions in which cartilaginous tumor tissue grew aggressively and caused entrapment of the trabecular bone, *i.e.*, the most important component to distinguish benign from malignant cartilaginous tumors [8–10]. Classical histopathology employs specific histological stains to highlight the cellularity of tumor tissue. The in-house developed hematein-based staining protocol enables the specific visualization of cell nuclei, and to a lesser extent the cytoplasm, within tumor cells.

While the feasibility of this methods was previously demonstrated using mouse liver tissue [12], to our knowledge, the presented study is the first to apply this methodology in a human tumor specimen. The staining properties of the hematein-based x-ray stain are caused by strengthening of the ionic interaction of the hematein lead (II) complex, which is positively charged, with the phosphate backbone of the deoxyribonucleic acid (DNA), which is negatively charged. Consequently, this results in the formation of a hematein lead (II) DNA complex, visualizing the cell nuclei as bright attenuation. In addition to cell nuclei, staining of the tumor tissue matrix was detected, which may be due to the binding of the positive charges of the hematein lead (II) complex to negatively charged proteoglycans within the cytoplasm of cells [23].

Tissue samples stained and prepared for micro-CT imaging were suitable for further histological investigations due to the nondestructive nature of the methodology, which is therefore compatible with routine diagnostic pathological investigations, such as H&E staining. It is possible to assess the conventional histology of the sample after applying the hematein-based x-ray stain, because decalcification of the tissue leads to the removal of the x-ray hematein-based stain [24]. In general, the nondestructive, high-resolution 3D microscopic recognition of tissue architecture may lead to a further, more detailed, assessment of certain widespread diseases in the future [25, 26].

When studying the matrix calcifications within the tumor tissue, the acidification step carried out as part of the x-ray hematein staining protocol did not influence the morphology of the sample. Also, acidification did not affect the visualization of the tumor calcifications, which play a major role in tumor classification and are especially present in cartilaginous tumors. In contrast, it is known that decalcification of the sample during histopathological preparation affects the sample morphology and that subtle calcifications may be removed by this procedure [27]. Therefore, calcifications are better visualized in the micro-CT images than in the histological slide, since the decalcification procedure for histology affects this diagnostically relevant feature.

Our study has certain limitations. First, to achieve the spatial resolution acquired in this feasibility study, the specimen size actually needed to be reduced to a cubic centimeter. Implementing the entire sample of the resected tumor in the workflow would mean an enormous impact for the method as well as for diagnosis. The final sample volume that can be stained *in vitro* still needs to be evaluated and even distribution of the stain throughout the sample most likely will limit the sample size. Nevertheless, constant modifications and improvements of *ex vivo* staining methods of tissue samples will allow the examination of samples larger than 1 cubic centimeter. With increasing the sample size, imaging of the entire resection specimen is feasible, yet the resolution voxel size would increase slightly. Second, only a small patient number (*n* = 3) was available and further studies are needed in order to confirm that x-ray stains in combination with micro-CT scanning are beneficial for the diagnosis of chondrosarcoma. Third, the equipment needed for sample preparation and the specific workflow requires a micro-CT, which is currently not available in all institutions. To make the workflow applicable, ideally each hospital should have a micro-CT either in the radiology or pathology department. This would allow for biopsies to immediately be processed and analyzed. Additionally, the time required for the whole workflow remains a limiting factor.

In conclusion, this study demonstrated the feasibility of the assessment of the 3D tissue architecture of tumor resection margins, specific radiological and histopathological diagnostic tumor features, and the potential value of the hematein-based x-ray staining method in combination with the high-resolution multi-slice micro-CT, providing unique nondestructive knowledge of chondrosarcoma at a microscopic level. Hematein-based staining may potentially be a suitable method for the diagnosis of chondrosarcoma in the clinical daily routine. Details of the 3D tissue architecture can be analyzed nondestructively helping to understand the nature and development of bone tumors.

### Supplementary Information

**Additional file 1.**

**Additional file 2: Supplementary Movie S1.** Animation illustrating the 3D volume shown in Fig. 2. Flythrough sequence of a chondrosarcoma sample stained with the hemateine-based X-ray staining.

**Additional file 3: Supplementary Movie S2.** Animation illustrating the 3D volume shown in Fig. 5. Flythrough sequence of a chondrosarcoma sample stained with the hemateine-based X-ray staining.

**Additional file 4: Supplementary Movie S3.** Animation illustrating the 3D volume shown in Fig. 6. Flythrough sequence of a chondrosarcoma sample stained with the hemateine-based X-ray staining.

## Data Availability

The data that support the findings of this study are available on request from the corresponding author ASG. The data are not publicly available due to them containing information that could compromise patient privacy.

## Abbreviations

3D: Three-dimensional
H&E: Hematoxylin and eosin
Micro-CT: Microscopic computed tomography
VOI: Volume of interest
