# Supplementary material for: Chondrosarcoma evaluation using hematein-based x-ray staining and high-resolution 3D micro-CT: a feasibility study
Source: Eur Radiol Exp. 2024 May 13;8:58. doi: 10.1186/s41747-024-00454-0 (PMC11089022; doi:10.1186/s41747-024-00454-0)
Supplement: Supplementary file 1 — Additional file 1. [file 41747_2024_454_MOESM1_ESM.pdf]

Chondrosarcoma evaluation using hematein-based x-ray staining and high-resolution 3D micro-CT: a feasibility study

ELECTRONIC SUPPLEMENTARY MATERIAL

Supplementary Figure 1

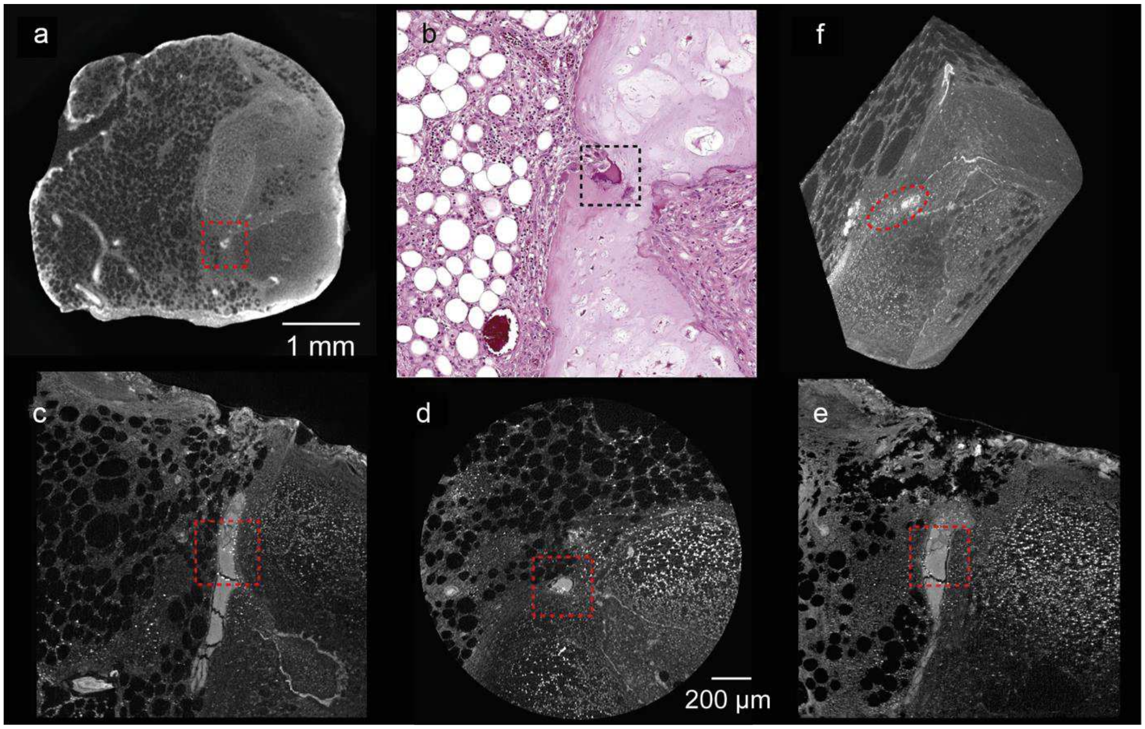

Supp. Figure 1. Trabecular entrapment. Hematein-based X-ray stain highlighted trabecular entrapment (rectangle) in 2D (a) and 3D volume rendering (f). 3D multiplanar reformation of the sagittal (c), axial (d) and coronal (e) plane of the labeled are from high-resolution microCT data. H&E-stained section of the corresponding layer (b).
